# Influence of Northern Wild Rice on Gut Dysbiosis and Short Chain Fatty Acids: Correlation with Metabolic and Inflammatory Markers in Mice on High Fat Diet

**DOI:** 10.3390/nu16172834

**Published:** 2024-08-24

**Authors:** Ruozhi Zhao, Janice Fajardo, Garry X. Shen

**Affiliations:** Departments of Internal Medicine, Food and Human Nutritional Sciences, University of Manitoba, Winnipeg, MB R3E 3P4, Canada; zhaorz@umanitoba.ca (R.Z.); janiceanne_13@hotmail.com (J.F.)

**Keywords:** wild rice, gut microbiota, fecal metabolites, mice, high-fat diet, metabolism, chronic inflammation, *Lactobacillus gasseri*, propionic acid

## Abstract

Wild rice (WLD) attenuated hyperglycemia, hyperlipidemia and chronic inflammation in mice receiving a high-fat diet (HFD) versus white rice (WHR), but the underlying mechanism is not well understood. We examined the influence of HFD + WLD on gut microbiota, short chain fatty acids (SCFAs) and the correlation with metabolic or inflammatory markers in mice versus HFD + WHR. C57BL/6J mice received HFD + 26 g weight (wt) % WHR or WLD or 13 g wt% WHR + 13 g wt% WLD (WTWD) for 12 weeks. Plasma levels of glucose, cholesterol and triglycerides, insulin resistance and inflammatory markers after overnight fasting were lower, and the abundances of fecal *Lactobacillus gasseri* and propionic acid were higher in HFD + WLD-fed mice than in HFD + WHR-fed mice. The anti-inflammatory effects of HFD + WTWD were weaker than HFD + WLD but were greater than those in HFD + WHR-fed mice. Abundances of fecal *Lactobacillus gasseri* and propionic acid in mice receiving HFD + WLD were higher than those in mice fed with HFD + WHR. The abundances of fecal *L. gasseri* and propionic acid negatively correlated with metabolic and inflammatory markers. The findings of the present study suggest that WLD attenuated metabolic and inflammatory disorders in mice on HFD. Interactions between WLD components and gut microbiota may upregulate fecal SCFAs, and the latter may be attributed to the benefits of WLD on metabolism and inflammation in mice on HFD.

## 1. Introduction

Northern wild rice (*Zizania palustris,* WLD) is an annual plant that naturally grows in shallow water in the Great Lakes and Canadian Prairie regions. WLD was a traditional food of Indigenous people in North America and it was known as Indian rice or manoomin [1]. WLD is not a typical kind of rice [2], but it has been used as a type of whole-grain product. WLD grain contains a significantly higher content of proteins, fiber, vitamins and antioxidants compared to refined white rice (WHR) [3], but the health benefit of WLD remains unclear.

Previous studies demonstrated that WLD reduced hypercholesterolemia, hypertriglyceridemia and the intensity of atherosclerosis in low-density lipoprotein receptor-deficient mice compared to WHR [4,5,6]. WHR is the most popularly consumed grain product worldwide. However, the refining process removes most nutrients from the outer layer of brown rice during the production of WHR. The intake of WHR as the major grain food is associated with increased risks for type 2 diabetes (T2D) [7,8]. T2D is characterized by hyperglycemia, insulin resistance, obesity, and chronic low-grade inflammation [9]. Our recent study demonstrated that the administration of a high-fat diet (HFD) supplemented with WLD resulted in lower fasting plasma glucose (FPG), cholesterol and triglycerides, insulin resistance and chronic inflammation compared to that in mice fed with HFD supplemented with the same dosage of WHR [10]. The underlying mechanism for the beneficial effects of WLD on metabolism and inflammation remains to be clarified.

The gut serves as the major organ to digest foods, absorb nutrients and play critical roles in metabolism and inflammation [11]. In addition, the gut contains the largest amount of bacteria in the body. The majority of bacteria present in the gut do not harm the body. Instead, many of them are beneficial to health since they may generate required nutrients from foods, modulate metabolism or inhibit inflammation or the overgrowth of pathological bacteria in the gut. The diversities and composition of gut microbiota were regulated by HFD [12]. The presence of gut bacteria is essential for HFD-induced obesity [13]. Multiple groups of bacteria promote the digestion of insoluble fiber from foods and generate short chain fatty acids (SCFAs) in the gut. SCFAs play active roles in the modulation of energy, metabolism and inflammation in the body [14]. The effect of WLD on the generation of the most abundant SCFAs in the gut and their relationship with the metabolism and inflammation in mice receiving HFD supplemented with WLD remains largely unknown.

The present study examined the micronutrients of WLD, the effects of the supplementation of two dosages of WLD on metabolism, vascular inflammation, proinflammatory cytokines, gut microbiota and fecal SCFAs in HFD-fed mice compared to WHR-supplementation and the correlations between fecal microbiota, SCFAs and circulatory metabolic or inflammatory cytokines in the mice.

## 2. Materials and Methods

### 2.1. Dietary Components

WLD was purchased from Floating Leaf Wild Rice Inc. in Springfield, MB. Asian white rice was obtained from a local grocery store. Carbohydrate-free HFD powder (D12492px11) was obtained from Research Diets (New Brunswick, NJ, Canada), which contains 47 g weight (wt) % of fat (91% from lard) and 35 g wt% of protein. Raw WLD and WHR were ground, and the particles were passed through a mesh with a pore size of 0.5 mm. After the supplementation of rice powder in 26 g wt%, the experimental diet contained 35 g wt% fat, accounting for 60% of the total calories, 26 g wt% of protein for 20% of calories and 26 g wt% carbohydrate for the remaining 20% of calories in the diet. The experimental diet was pelleted and stored at −20 °C before the start of feeding.

### 2.2. Experimental Animals

Male C57 BL/6 J mice (6 weeks of age) were purchased from the Jackson Laboratory (Bar Harbor, ME, USA). Mice were hosted in an air-conditioned room in stainless steel cages. They received regular rodent chow for one week after arrival. The mice were randomly divided into 3 groups (*n* = 5/group) before the start of the regimen. Mice in the WHR diet group received HFD supplemented with 26 g wt% of WHR (*w*/*w*). The WTWD diet group was fed with HFD supplemented with 13 g wt% of WHR and 13 g wt% of WLD. The WLD diet group received HFD supplemented with 26 g wt% of WLD. The dietary intervention lasted for 12 weeks.

### 2.3. Animal Monitoring and Sample Collection

Body weights and daily food intake were recorded prior to the onset and at the end of the regimen. Blood was withdrawn from the saphenous vein to analyze biochemical variables after an overnight fasting. At the end of the regimen, mice were euthanized between 10 am and 1 pm via isoflurane (5%, *v*/*v*) inhalation. Abdominal aortae were harvested and submerged in a culture dish containing Hank’s balanced salt solution (HBSS) on ice for ex vivo monocyte adhesion assays, as previously described [15]. Feces were collected from cages of individually hosted mice and stored at −80 °C. The protocol for animal experiments was approved by the Animal Management and Protocol Committee at the University of Manitoba.

### 2.4. Measurements of Circulatory Glucose, Triglycerides and Cholesterol

The levels of plasma glucose, total cholesterol and triglycerides of mice after an overnight fasting were analyzed by enzymatic methods using reagent kits from Sekisui Diagnostics (Charlottetown, PE, Canada). Glucose was analyzed using the glucose-6-phsphate dehydrogenase method (cat. #235-60). Cholesterol was measured using the hydrogen peroxidase method (cat. #234-60) and triglycerides were assessed using the glycerol phosphate oxidase method (cat. #236-60) following the manufacturer’s instructions.

### 2.5. Measurements of Plasma Insulin and Pro-Inflammatory Cytokines

The levels of insulin and pro-inflammaoty cytokines, tumor necrosis factor-α (TNFα), plasminogen activator inhibitor-1 (PAI-1) and monocyte chemotactic protein-1 (MCP-1) antigens in plasma were analyzed using enzyme-linked immunosorbent assay (ELISA) kits. For mouse insulin, the kits were obtained from EMD Millipore (Billerica, MA, USA for insulin). For mouse TNFα, kits were obtained from BD Bioscience (San Diego, CA, USA). For mouse MCP-1, the kits were received from Thermo Scientific (Ottawa, ON, Canada). For mouse PAI-1, the kits were obtained from Oxford Biomedical Research (Oxford, MI, USA). Homeostatic model assessment-insulin resistance (HOMA-IR) was calculated from plasma glucose and insulin in simultaneously collected blood samples using a mouse-specific formula [16].

### 2.6. Monocyte Adhesion Assay

Fluorescently labeled WEHI-274.1 mouse monocytes (1 *×* 10^5^) in 1 mL of RPMI 1640 medium were added to each dish containing one strip of aorta and incubated at 22 °C for 0.5 h on a horizontally rotating mixer. Non-adhered monocytes were removed via two washes using ice-cold HBSS. Monocytes adhered on aortic intima were counted under microscopy (10 X magnification). The average counts of monocytes adhered on the intima of one aorta from five independent fields were applied in data analysis [15].

### 2.7. Extration of Mouse Fecal Bacteria DNA and 16S rRNA Gene Sequencing

Mouse fecal DNA was extracted using the Qiagan Power Fecal DNA Isolation Kit (Germantown, MD, USA) and quantified using a NanoDrop spectrophotometer (Thermo Scientific). Fecal DNA was amplified using a pair of primers targeting the V4-V5 region of the bacterial DNA sequence [515F (5′-GTGYCAGCMGCCGCGGTAA) and 926R (5′-CCGYCAATTYMTTTRAGTTT)]. The quality of PCR products was verified using analytical gels. Unqualified amplicons were repeatedly amplified with modifying PCR conditions until qualified bands were obtained. DNA amplicons were normalized using Charm Biotech 96-well normalization kit, and 16S rRNA gene sequencing was conducted via an Illumina MiSeq sequencer at the Integrated Microbiome Resource at Dalhousie University [17].

### 2.8. Bioinformatics Analyses of Gut Microbiota

The raw gut microbiome data were trimmed to remove primers. Trimmed reads were imported into Quantitative Insights on the Microbial Ecology 2 (QIIME2) platform using QIIME 2 (2023.2 version). Diversity metrics in QIIME2 were used to evaluate α- and β-diversity [17]. Liner discriminant analysis Effect Size (LEfSe) of gut microbiota was analyzed using the Galaxy module at https://galaxy.ansible.com/ui/, accessed on 12 March 2023

### 2.9. Analysis of Fecal SCFAs

Fecal samples (50–150 mg) were mixed with 1 mL of 0.006 M NaOH and homogenized for 10 min. After centrifugation (13,200× *g*) for 10 min at 4 °C, the supernatant was collected. Fatty acids were extracted using 0.5 mL of propanol and pyridine (3:2, *v*/*v*) and derivatized using propyl chloroformate. The analysis of SCFAs was conducted on an Agilent 7890A gas chromatography coupled with 5975A mass spectrometry at Microbiome Insights Inc. (Vancouver, BC, Canada), as described in [18].

### 2.10. Extraction and Metabolomics Sample Analysis of WLD

Rice powders (~100 mg) were milled and passed through a 60 mesh and then extracted with 2 mL of CH_3_OH: H_2_O (3:2, *v*/*v*). The mixture was vortexed for 2 min and sonicated for 40 min at 4 °C. After centrifugation (14,000 rpm) for 10 min at 4 °C, the supernatant was dried under nitrogen. Dried extracts were reconstituted with 0.2 mL of solvent containing water/acetonitrile (4:1, *v*/*v*) and 300 ng norvaline. For each sample, 2 μL of extract was injected with a flow rate of 0.7 mL/min. Metabolomics analyses were performed in a high-performance liquid chromatography system coupled with a 6538 UHD Accurate LC-Quadrupole Time-Of-Flight-mass spectrometry (QTOF-MS) (Agilent Technologies, Santa Clara, CA, USA) with a dual electrospray ionization (ESI) source. A 2.1 mm × 100 mm Agilent ZORBAX SB-Aq column (Agilent Technologies) was maintained at 60 °C for chromatographic separation of samples using water (A) and acetonitrile (B) containing 0.1% formic acid. The reaction running time was 10 min with a gradient of 0 ± 6 min 2% B; 6 ± 8.50 min 60% B; 8.50 ± 8.60 min 2% B and 8.60 ± 10 min 2% B.

### 2.11. Metabolomics Data Acquisition

Metabolomic data acquisitions were accomplished in positive (+) and negative (−) ESI modes. MS spectra were collected in the range of 50 ± 1700 *m*/*z* with known reference masses of 121.0508 and 922.0097 (ESI+) and 112.9860 and 1033.9880 (ESI−) during all runs. QTOF-MS data were analyzed using multiple algorithms incorporated in Agilent Mass Hunter Qualitative (version 7.01) and Mass Profiler Professional software(version 2021) [10]. The Kyoto Encyclopedia of Genes and Genomes database was used to assign potential physiological functions.

### 2.12. Statistical Analyses

Probabilities from data >2 groups were analyzed using the one-way analysis of variance assay (ANOVA), followed by the Kruskal–Wallis test paired with post-hoc Tukey test or Pairwise Wilcoxon test for probability between two groups. Correlations between two sets of variables were analyzed using linear regression analysis. OriginPro 2021 software (version 2021.2) was used for plotting and statistical analysis. Quantitative data were expressed in means ± standard deviations (SDs) and a *p* < 0.05 was considered as statistically significant.

## 3. Results

### 3.1. Macronutrients and Metabolomic Metabolites in WLD versus WHR

Based on the data from the U.S. Department of Agriculture, 100 g of raw WLD contains similar calories and carbohydrates as 100 g of long strain WHR. However, WLD contains >2-times the amount of protein and >4-times the amount of fiber compared to long strain WLR (Table 1) [19,20].

**Figure 1:**
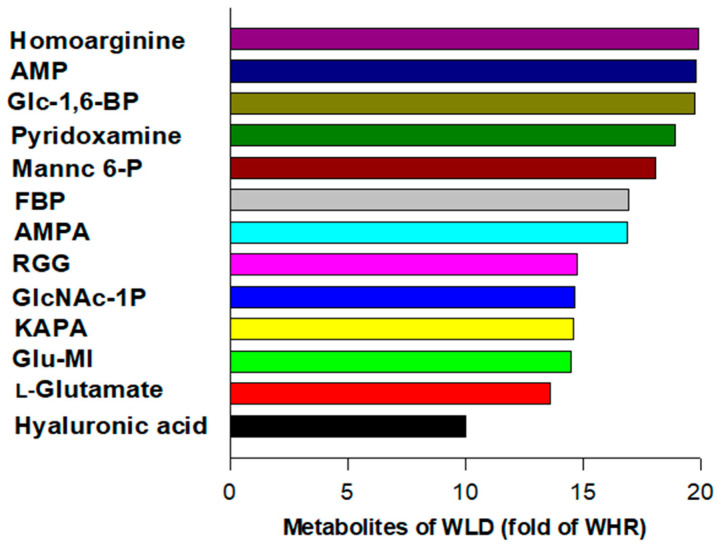
Major metabolites in wild rice (WLD) compared to white rice (WHR). Fold increase in metabolites related to glucose metabolism and diabetes in WLD versus WHR detected by HPLC-QTOF-MS. AMP: adenosine 5-monophosphate; Glc-1-6-BP: α-D-glucose 1; 6-phophate; mannc 6-P: *N*-Acetyl-D-mannosamine 6-phosphate; Glu-MI: 6-(β-D-glucosaminyl)-1D-myo-inositol; RGG: α-L-rhamnopyranosyl-(1-2)-β-D-galactopyranosyl-(1)-β-D-glucopyranoside; AMPA: aminomethylphosphonic acid; KAPA: 8-amino-7-oxononanoate.

The present study detected 1892 metabolites in WLD and WHR via metabolomic analysis. A group of metabolites was 10-20-fold more abundant in WLD than in WHR (*p* < 0.001), which potentially regulates glucose metabolism, insulin, vascular tone, or inflammation or has known therapeutic effects for diabetes or its complications (Figure 1). The metabolites were listed decently according to their intensities in fold increases in their relative abundances in WLD compared to WHR, which include amino acids and nucleotides (homoarginine, L-glutamate, adenosine 5-monophosphate or AMP), carbohydrates [α-D-glucose 1, 6-phophate or Glc-1-6-BP, *n*-Acetyl-D-mannosamine 6-phosphate or mannc 6-P, hyaluronic acid o 6-(β-D-glucosaminyl)-1D-myo-inositol or Glu-MI] or other natural products with capacities for modulating metabolic or inflammatory pathways [pyridoxamine, α-L-rhamnopyranosyl-(1-2)-β-D-galactopyranosyl-(1)-β-D-glucopyranoside (RGG), aminomethylphosphonic acid (AMPA) and 8-amino-7-oxononanoate (KAPA)].

### 3.2. Effects of WLD on Glucose, Lipids and HOMA-IR in HFD-Fed Mice

**Figure 2:**
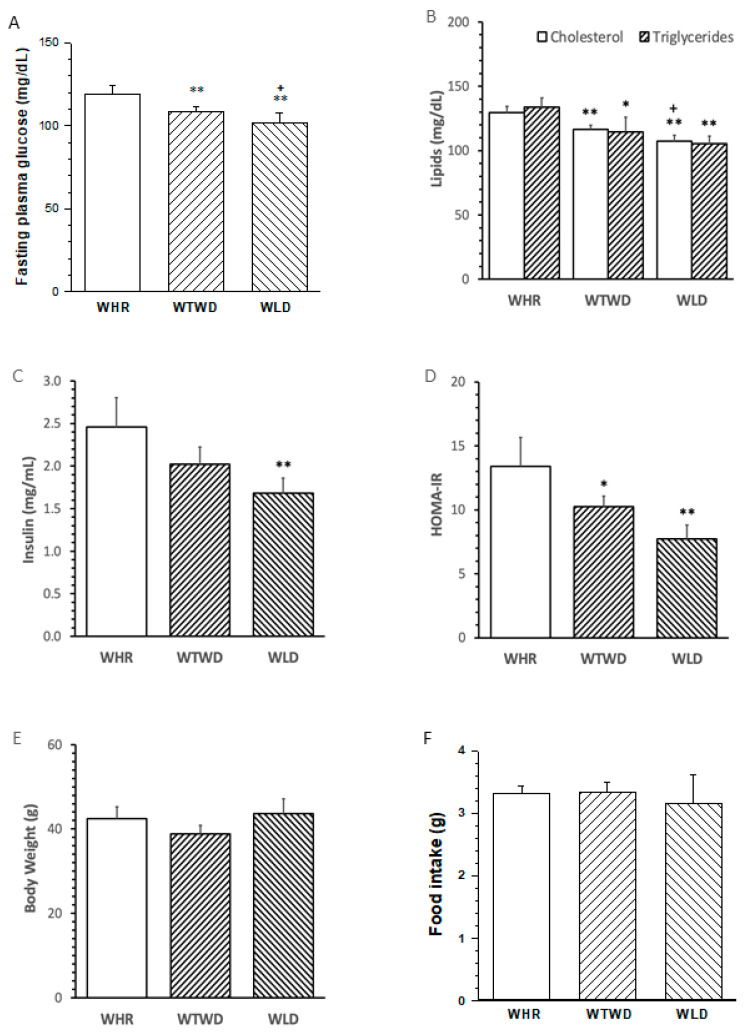
Influence of WLD and WHR on metabolism in HFD-fed mice. Male C57 BL/6 J mice were fed with HFD supplemented with 26 g wt% of WHR, 26 g wt% of WLD or 13 g wt% of WHR + 13 g wt% of WLD (WTWD) for 12 weeks. Blood samples were withdrawn via saphenous vein after an overnight fasting for the measurements of the levels of fast plasma glucose (FPG), total cholesterol, triglycerides and insulin. Body weights were measured on the day before tissue harvesting. Food intake was assessed for the 24 h period during the last week of the experiment. (**A**): FPG. (**B**): total cholesterol and triglycerides. (**C**): insulin; (**D**): homeostatic model assessment-insulin resistance (HOMA-IR); (**E**): body weight. (**F**): daily food intake. The values were expressed in mean ± SD mg/dL (*n* = 5/group) or gram (g). *, **: *p* < 0.05 or 0.01 versus WHR group (ANAVA, Tukey test); +: *p* < 0.05 versus WTWD group.

Fasting plasma glucose (FPG) levels in mice receiving HFD supplemented with 26 g wt% WLD (WLD diet) or 13 g wt% WLDL + 13 g wt% WHR (WTWD det) for 12 weeks were significantly lower than that in mice fed with HFD + 26 g wt% WHR (WHR diet, *p* < 0.01 or 0.05, Figure 2A). The levels of total cholesterol and triglycerides in mice treated with the WLD diet or WTWD diet were lower than those in mice who received the WHR diet (*p* < 0.05 or 0.01, Figure 2B). The WLD diet and dose-dependent WTWD diet reduced fasting plasma insulin and the HOMA-IR versus WHR diets (Figure 2C,D). No significant difference in body weights or food intake (Figure 2E,F) was detected among the mice receiving the HFD diet supplemented with three different types of rice.

### 3.3. Effects of WLD on Circulatory Inflammatory Cytokines and Monocyte Adhesion

**Figure 3:**
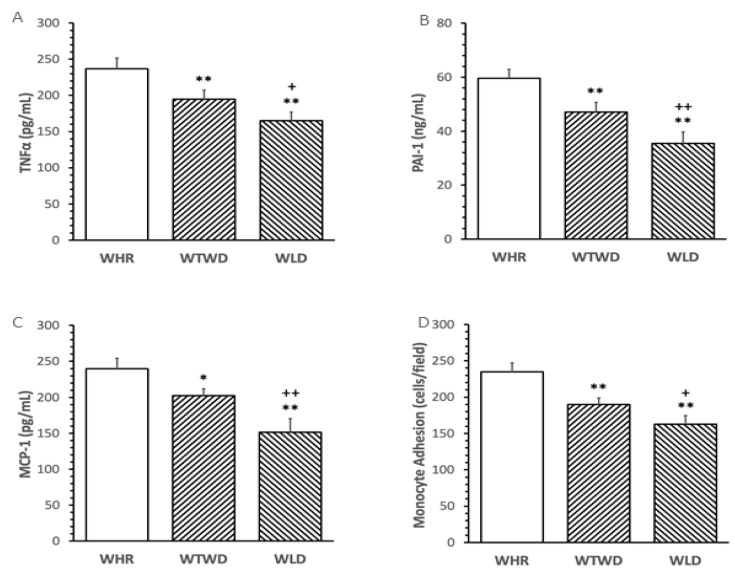
Effects of WLD and WHR on inflammatory cytokines and monocyte adhesion in HFD-fed mice. The regimen for the dietary intervention and animal groups was identical to that described in Figure 2. Blood was collected from mice at the end of the regimen. (**A**–**C**): plasma levels of tissue necrosis factor-α (TNFα), monocyte chemotactic protein-1 (MCP-1) and plasminogen activator inhibitor-1 (PAI-1) were measured using ELISA. Values were expressed in the mean ± SD pg/mL or ng/mL (*n* = 5/group). (**D**): monocyte adhesion to the aorta was assessed as described in the Methods and expressed in cells/field (*n* = 5/group). Blank bar: WHR, stripped right-to-lef: WTWD; stripped left-to-right WLD. *, **: *p* < 0.05 or 0.01 versus WHR group; +, ++: *p* < 0.05 or 0.01 versus WTWD diet (ANOVA, Tukey test).

Supplementation of WLD dose-dependently reduced the levels of pro-inflammatory cytokines, PAI-1, MCP-1 and TNFα in plasma and ex vivo monocyte adhesion to the intima of mouse aortae in comparison to that from HFD + WHR-fed mice (*p* < 0.01, Figure 3A–C). The WLD diet induced significantly greater inhibition of the pro-inflammatory mediators and monocyte adhesion compared to that of mice receiving the WTWD diet (*p* < 0.01, Figure 3D).

### 3.4. Influence of WLD on Diversities of Gut Microbiota

**Figure 4:**
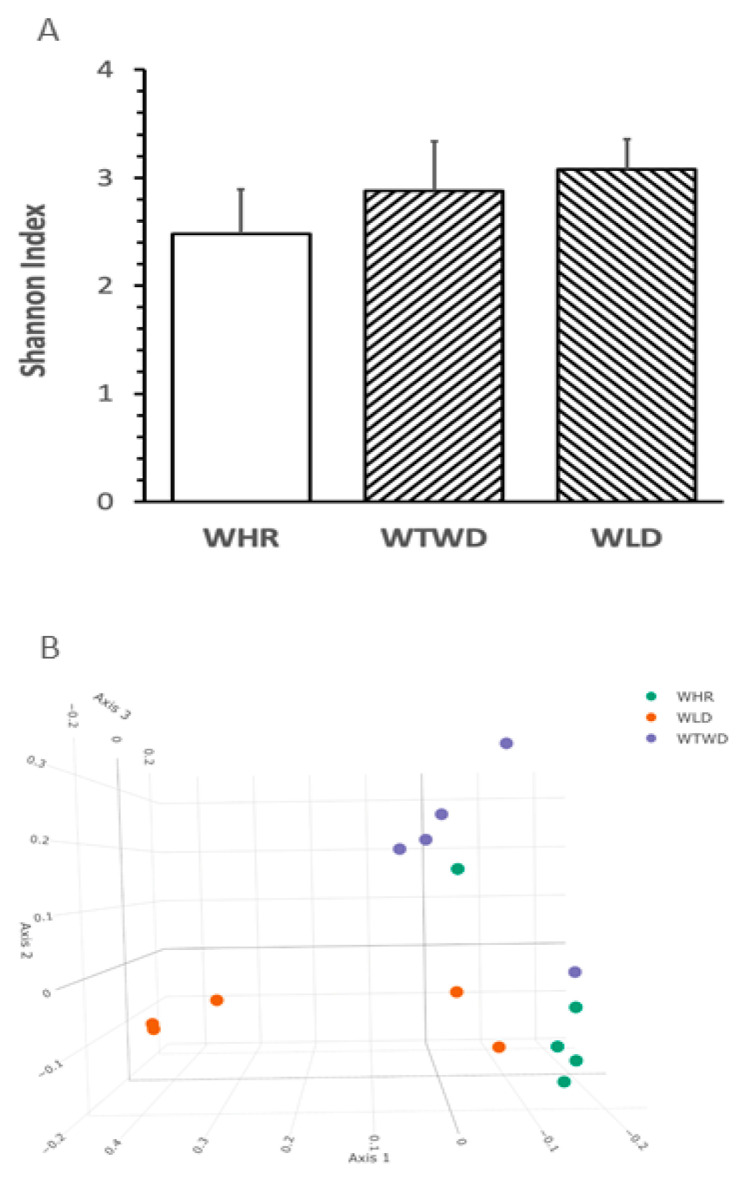

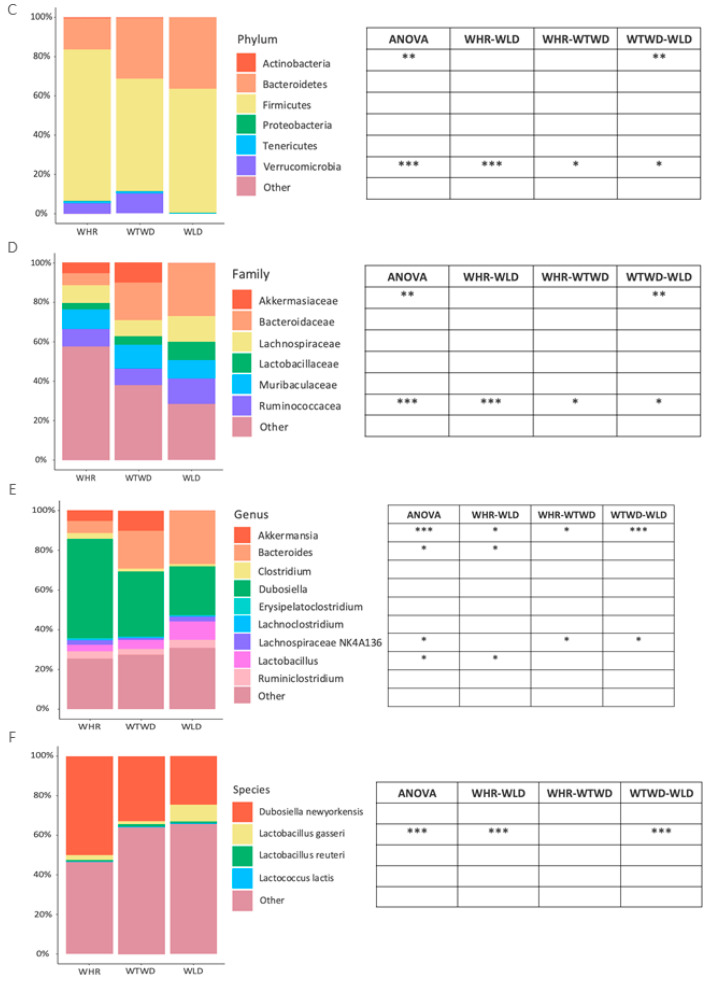

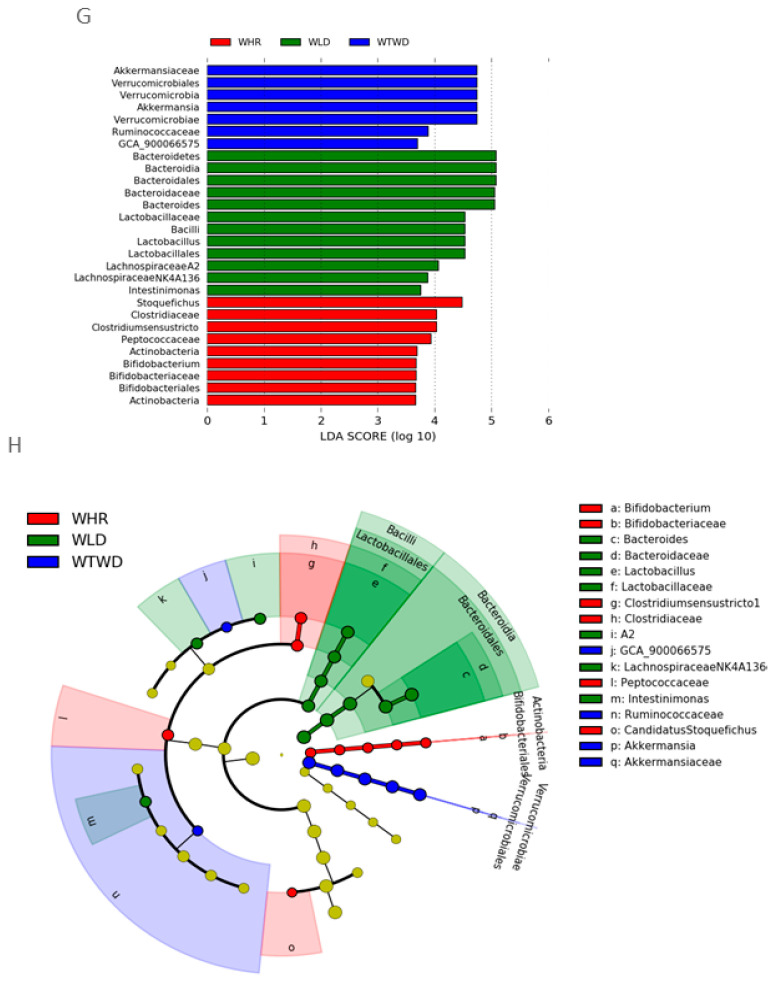
Effects of WLD and WHR on taxonomy of gut bacteria in mice receiving HFD. The regimen for the dietary intervention and animal groups was identical as described in Figure 2. Stool samples were harvested from mice individually housed in cages before the end of the regimen. Gut microbiota was analyzed using 16S rRNA gene sequencing. (**A**): Shannon index of gut microbiota (mean ± SD, *n* = 5/group); (**B**): PCA plot for β-diversity based on Bray–Curtis dissimilarities (weighted by taxon abundance). Effects of WLD and WHR on taxonomy of gut microbiota in HFD-fed mice. The design of the dietary regimen was identical in Figure 2 and the procedure for 16S-rRNA sequencing was the same as described in Figure 4A,B. Relative abundances of major types of bacteria in various taxonomies were presented on the left of the figures and statistical analysis results between groups were listed in the tables on the right. (**C**): phylum bacteria; (**D**): family bacteria; (**E**): genus bacteria; (**F**): species bacteria. Blank bar: WHR, stripped right-to-lef: WTWD; stripped left-to-right WLD. *, **, ***: *p* < 0.05, 0.01 or 0.001 (ANOVA, Tukey test). Effects of WLD and WHR on taxonomy of gut microbiota in HFD-fed mice. The regimen for the dietary intervention and animal groups was the same as that in Figure 2 and the procedure for 16S-rRNA sequencing was identical to that in Figure 4A,B. The linear discriminant analysis effect size (LEfSe) of fecal bacteria in HFD-fed mice supplemented with different types or doses of rice indicates the types of rice that were conducted using the Galaxy module. (**G**): liner discriminant analysis. (**H**): cladogram.

No significant difference in the levels of Shannon index, a common variable used for assessing richness and evenness of α-diversity in gut microbiota, was detected in feces from the three groups of mice receiving different dietary interventions (Figure 4A). The plot of principal component analysis demonstrated that the β-diversity in HFD + WLD diet-fed mice was well separated from that of HFD + WHR-fed mice and that in HFD + WTWD-fed mice partially overlapped with that of HFD + WHR-fed mice as expected (Figure 4B).

The most common fecal phylum bacteria of all three groups of mice were *Firmicutes* and *Bacteroidetes*, but no significance was detected in the relative abundances between the two predominant phylum bacteria in feces among the three groups of mice receiving HFD supplemented with different types or doses of rice. The abundances of *Actinobacteria* phylum bacteria in mice receiving the WTWD diet were significantly lower than that receiving the WLD diet (*p* < 0.01), but no significant difference was detected between the groups of mice receiving the WHR versus WLD or the WHR versus WTWD diet. The abundances of *Vernrucomicrobia* phylum bacteria in the WLD diet-fed mice were lower than that in the WHR or WTWD diet-fed mice (*p* < 0.05 or 0.001) and that in mice receiving the WTWD diet were higher than that receiving the WHR diet (*p* < 0.05, Figure 4C). The abundance of fecal *Akkermansiaceae* family of bacteria in the WTWD diet-fed mice was higher than that in mice receiving the WLD diet (*p* < 0.01). The abundance of the *Ruminococcaceae* family of bacteria in the WLD diet-fed mice was higher than that in the WHR or WTWD diet-fed mice (*p* < 0.05 or 0.001). The abundance of *Ruminococcaceae* in the WTWD diet-fed mice was lower than that in mice receiving the WHR diet (*p* < 0.05, Figure 4D). The abundance of *Akkermansia* genus bacteria in mice on the WLD diet was lower than that in mice receiving the WHR or WTWD diet (*p* < 0.05 or 0.001). The abundance of fecal *Bacteroides* and *Lactobacillus* genus bacteria in the WLD-fed mice was higher than that in mice receiving the WHR diet (*p* < 0.05). The abundance of *Lachnospiraceae NK4A136* genus bacteria in the WTWD diet-fed mice was lower than that in mice receiving the WHR or WLD diet (*p* < 0.05, Figure 4E). The abundance of fecal *Lactobacillus gasseri* (*L. gasseri*) species of bacteria in mice receiving the WLD diet was higher than that in mice on the WHR or WTWD diet (*p* < 0.001, Figure 4F).

The results of the LEfSe analysis displayed distinct patterns of bacteria in the gut of mice receiving HFD + WHR, WLD or WTWD. The gut bacteria profile of mice receiving the WHR diet was characterized by the enrichment in *Bifidobacterium* phylum, *Bifidobacteriaceae, Clostridiaceae* and *Peptococaceae* family of bacteria. The WLD diet-fed mice were highlighted by *Bacteriodaceae, Lactobacillaceae* family bacteria and *Bacteroides* and *Lactobacillus* genus bacteria. The mice receiving the WTWD diet were featured by *Verruconicrobia* phylum, *Ruminococaceae* family, *Akkermansia* family and genus bacteria (Figure 4G,H).

### 3.5. Impact of WLD on SCFAs and Relationship with Metabolic or Inflammatory Variables

**Figure 5:**
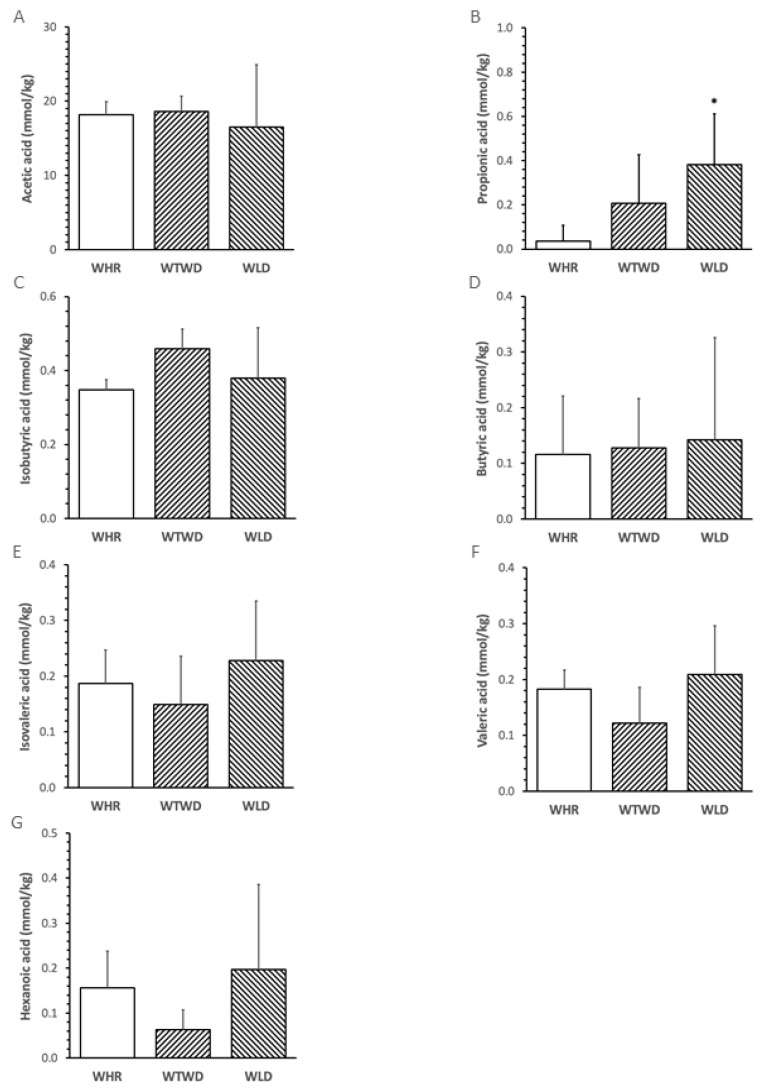
Effects of WLD and WHR on abundances of short chain fatty acids (SCFAs) in feces of HFD-fed mice. The regimen of dietary intervention and animal groups was the same as described in Figure 2. SCFAs were analyzed using GC-MS. (**A**): acetic acid; (**B**): propionic acid; (**C**): isobutyric acid; (**D**): butyric acid; (**E**): isovaleric acid; (**F**): valeric acid; (**G**): hexanoic acid. Values were presented in mean ± SD mmol/kg feces (*n* = 5/group). Blank bar: WHR, stripped right-to-lef: WTWD; stripped left-to-right WLD. *: *p* < 0.05 versus WHR diet (ANOVA, Tukey test).

**Figure 6:**
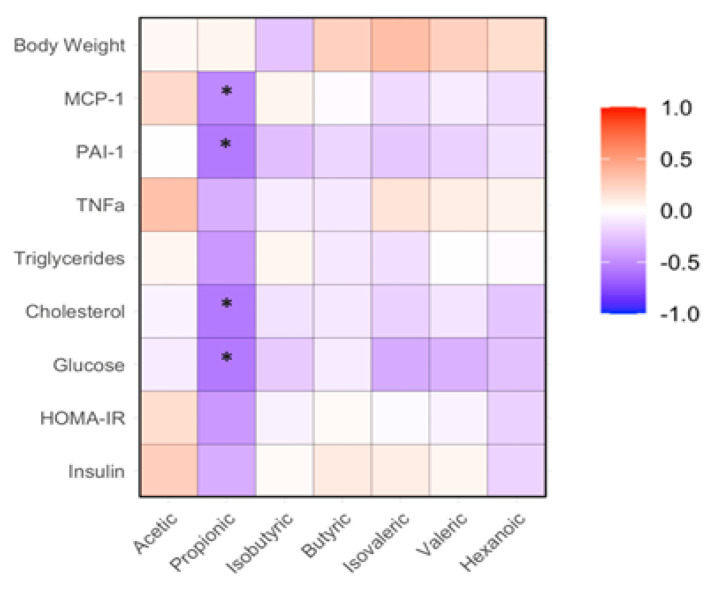
Correlation between SCFAs and metabolic or inflammatory markers in mice receiving HFD supplemented with WLD or WHR. The regimen was identical to that of Figure 2. SCFA measurements were conducted as described in the Methods. *: *p* < 0.05 between SCFA and biochemical variable using linear regression analysis (*n* = 15).

The abundance of fecal propionic acid in mice receiving the WLD diet was significantly higher than that in mice receiving the WHR diet (*p* < 0.05, Figure 5B). No significant difference in the abundances of other fecal SCFAs was detected among the three groups (Figure 5A,C–G). The abundance of fecal propionic acid negatively correlated with the levels of FPG, total cholesterol, PAI-1 and MCP-1 in the plasma of the mice (*p* < 0.05, Figure 6).

### 3.6. Correlation between Fecal SCFAs and Gut Bacteria

**Figure 7:**
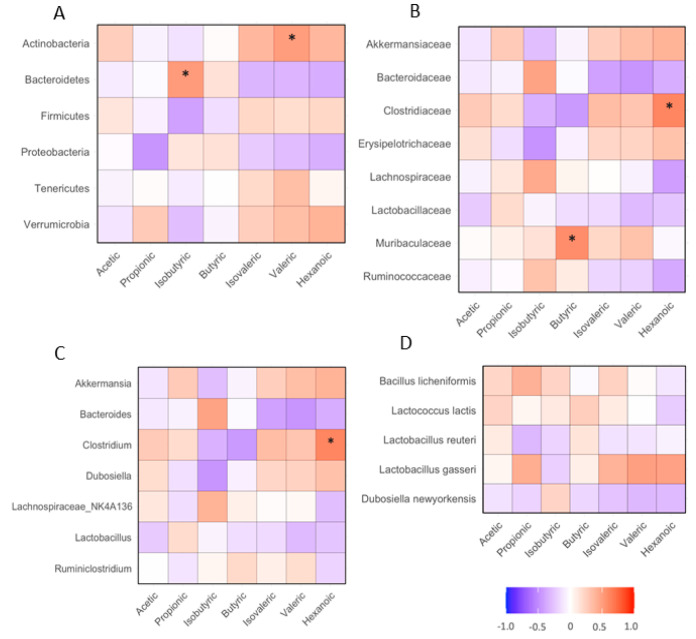
Correlation between fecal SCFAs and gut microbiota in HFD-fed mice. The design of the dietary regimen and animal groups was identical, as described in Figure 2. Bacterial gene sequencing and SCFAs analysis were conducted as described in the Methods. (**A**): correlation between SCFAs and phylum bacteria; (**B**): correlation between SCFAs and family bacteria; (**C**): correlation between SCFAs and genus bacteria; (**D**): correlation between SCFAs and species bacteria. *: *p* < 0.05 between SCFA and biochemical variable using linear regression analysis (*n* = 15).

The abundance of fecal isobutyric acid positively correlated with *Bacteroidetes* phylum bacteria in the gut (*p* < 0.05). The abundance of fecal valeric acid positively correlated with fecal *Actinobacteria* phylum bacteria (*p* < 0.05, Figure 7A). The abundance of fecal butyric acid positively correlated with the fecal *Muribaculaceae* family of bacteria (*p* < 0.05). The abundance of fecal hexanoic acid positively correlated with the fecal *Clostridiaceae* family of bacteria (*p* < 0.05, Figure 7B). The abundance of fecal hexanoic acid positively correlated with the fecal *Clostridum* genus bacteria (*p* < 0.05, Figure 7C). The abundances of SCFAs did not significantly correlate with any type of species of bacteria in the mice (Figure 7D).

### 3.7. Correlation between Fecal and Body Weights, Metabolic or Inflammatory Markers

**Figure 8:**
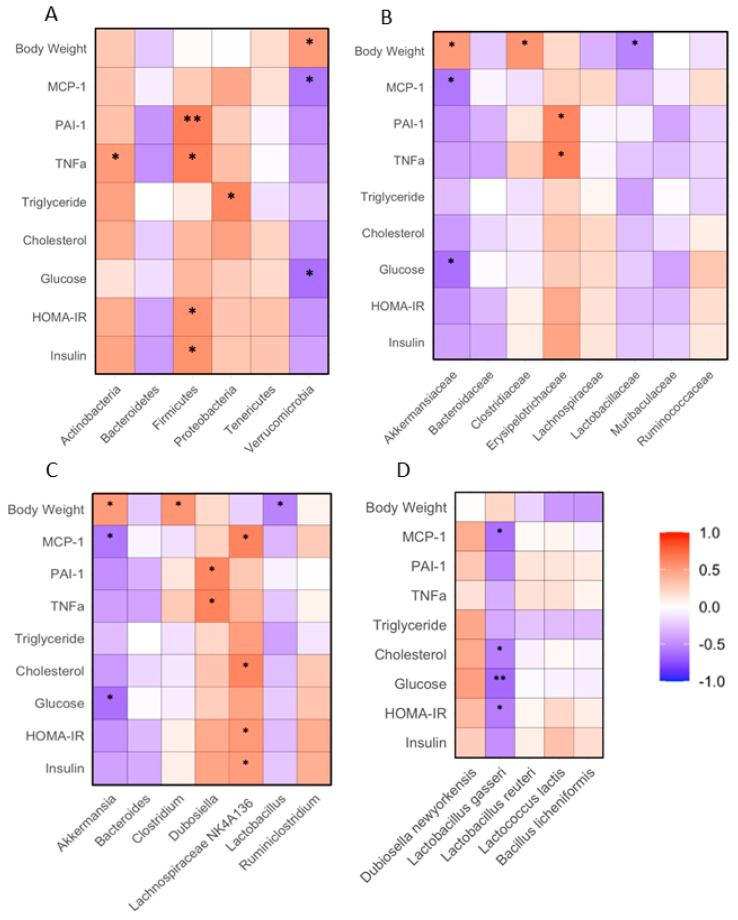
Correlation between gut microbiota and biochemical variables in HFD-fed mice. The regimen of dietary intervention and animal groups was the same as that in Figure 2. Bacterial gene sequencing, biochemical analysis and body weights were conducted as described in the Methods. (**A**): Correlation between phylum bacteria, biochemical variables or body weights; (**B**): correlation between family bacteria, biochemical variables or body weights; (**C**): correlation between genus bacteria, biochemical variables or body weights; (**D**): correlation between species bacteria, biochemical variables or body weights. *, **: *p* < 0.05 or 0.01 between gut bacteria and biochemical variable or body weight using linear regression analysis (*n* = 15).

The relative abundance of fecal *Firmicutes* positively correlated with insulin, HOMA-IR, TNFα and PAI-1 in the mice receiving the HFD supplemented with WHR, WTWD or WLD (*p* < 0.05 or 0.01). The abundance of fecal *Verrucomicrobia* negatively correlated with FPG and MCP-1, while it positively correlated with the body weight of the mice (*p* < 0.05, Figure 8A). The abundance of fecal *Akkermansiaceae* family of bacteria negatively correlated with FPG and MCP-1 but positively correlated with the body weights of the mice (*p* < 0.05). The abundance of fecal *Erysipelotrichaceae* family of bacteria positively correlated with PAI-1 and TNFα (*p* < 0.05, Figure 8B). The abundance of fecal *Akkermansia* genus bacteria negatively correlated with FPG and MCP-1 and positively correlated with the body weights of the mice (*p* < 0.05). The abundance of *Dubosiella* genus bacteria positively correlated with PAI-1 and TNFα levels. The abundance of fecal *Lachnosipiraceae NK4A136* genus bacteria positively correlated with insulin, HOMA-IR, cholesterol and MCP-1 (*p* < 0.05, Figure 8C). The abundance of fecal *L. gasseri* negatively correlated with HOMA-IR, FPG, cholesterol and MCP-1 of the mice (*p* < 0.05, Figure 8D).

## 4. Discussion

The results of the present study demonstrated that the WLD dose-dependently attenuated circulatory metabolic and pro-inflammatory markers in the HFD-fed mice compared to those receiving HFD + WHR. The relative abundance of probiotic *L. gasseri* species of bacteria in the feces of mice receiving HFD + WLD was significantly greater than that in mice receiving HFD + WHR or HFD + WTWD. The abundance of fecal propionic acid in the HFD + WLD-fed mice was significantly higher than in the HFD + WHR-fed mice. The fecal abundances of both *L. gasseri* and propionic acid negatively correlated with cholesterol, pro-inflammatory cytokines, FPG or HOMA-IR in mice. Metabolomics analysis indicated that WLD contained substantially higher abundances of metabolites with the capacity to modulate glucose metabolism compared to WHR, including AMP, homoarginine, Glc-1, 6-BP, Glu-MI and L-glutamate.

Previous studies by Moghadasian et al. demonstrated that WLD treatment-induced attenuation of atherosclerosis was associated with increased abundances of fecal bacteria, including *Lactobacillus* and of fatty acids containing four or more carbons but not the most abundant SCFA, acetic acid or propionic acid, in LDL receptor-deficient mice [21]. Hou et al. reported that Asian wild rice (Zizania latifolia) intake reduced liver steatosis, insulin resistance and gut dysbiosis in mice fed with HF diet [22]. The present study for the first time examined the effects of North American WLD on fecal content, including acetic acid and propionate acid, in addition to 4–6 SCFAs, and on the correlation between fecal SCFAs and bacteria in HFD-fed mice. The findings of the present study demonstrated that WLD significantly increased the abundance of fecal propionic acid, and the abundance of fecal propionic acid negatively and significantly correlated with multiple metabolic and inflammatory markers in HFD-fed mice.

The results of LEfSe analysis demonstrated that the feces of the WLD diet-fed mice were enriched with the *Lactobacillaceae* family and *Lactobarcillus* genus bacteria. The abundance of fecal *L. gasseri* species of bacteria in mice fed with the WLD diet was significantly higher than that in WHR or WTWD diet-fed mice. Although no significant correlation was detected between the abundance of fecal *L. gasseri* and propionate acid in the mice, both *L. gasseri* and propionate acid negatively correlated to FPG, cholesterol and MCP-1 in the mice, which suggests close associations between gut bacteria, SCFA and metabolic and inflammatory variables in the HFD-fed mice.

Several species of *Lactobacillus*, including *L. gasseri*, have been recognized as potent probiotics for the management of diabetes and obesity in clinical trials [23,24]. The findings of the present study suggest that the increases in the abundance of *L. gasseri* in the gut may subsidize the metabolic and anti-inflammatory benefits of WLD in mice on a Western type of diet. The relationship between *L. gasseri* and the production of propionate acid in the gastrointestinal tract is warranted to be investigated.

In addition, the abundances of *Bacteroides* genus bacteria and *Ruminococcaceae* family of bacteria in the feces of mice receiving HFD + WLD were significantly higher than that in mice receiving HFD + WHR. The abundance of fecal isobutyric acid positively correlated with *Bacteroidetes* phylum bacteria in the mice, but there was no detectable correlation between fecal isobutyric acid and metabolic or inflammatory markers in the mice.

Other components of WLD may be also implicated in the metabolic and anti-inflammatory regulation in HFD-fed mice. For example, WLD contains 4–5-times higher content of fiber compared to WHR [19,20]. Previous studies by other groups demonstrated that a diet enriched with fiber reduces glucose intolerance and insulin resistance in T2D patients, which was associated with an enhancement of a variety of gut bacteria capable of generating SCFAs and increased circulatory levels of glucagon-like peptide-1 [25,26]. Our group previously demonstrated that fiber-rich brown rice and germinated brown rice attenuated glucose, lipids and pro-inflammatory cytokines in HFD-fed mice [13]. The precise role of fiber in the beneficial effects of WLD, brown or germinated brown rice, on metabolism, inflammation, gut microbiota and SCFA production are warranted to be further investigated in subsequent studies.

WLD contains nearly 20-fold higher levels of AMP compared to WHR. AMP is a known agonist of AMP-activated protein kinase (AMPK) and the latter plays vital roles in the regulation of the metabolism of glucose and lipids, insulin resistance and inflammation [27]. A previous study demonstrated that the colonization of *Lactobacillus rhamnosus* upregulated the phosphorylated AMPK in the colon of mice [28]. Although the concentration of AMP in WLD was not quantified in the present study, a previous study by our group demonstrated that the supplementation of 26 g wt% of WLD in the HFD diet increased the abundance of phosphorylated AMPKα in the liver, skeletal muscle and adipose tissue compared to that in mice receiving HFD supplemented with an equal dosage of WHR in mice [10]. The finding suggests that AMP in the dosage of WLD equivalent to that in the present study is able to activate the AMPKα in insulin-sensitive tissue in mice.

Propionic acid is a precursor of liver gluconeogenesis [29]. A previous study indicated that propionic acid reduced gluconeogenesis in HepG2 hepatocytes via the activation of the AMPK pathway [30]. Propionic acid inhibited insulin-induced de novo lipogenesis and increased glucose uptake in primary rat adipocytes [31]. Treatment of human adipose tissue explants with propionic acid resulted in downregulations of inflammatory cytokines and upregulations of lipoprotein lipase and glycose transporter [32]. Propionic acid also improved diabetes-induced endoplasmic reticulum stress in rat ventromedial hypothalamus [33]. In addition, WLD also contains abundant amounts of L-glutamate [34], AMPA [35], KAPA [36,37], homoarginine [38,39], Glc-1 and 6-BP [40,41], which have known biological involvements in the regulation of glucose metabolism or insulin resistance. Pyridoxamine or vitamin 6 [42,43], RGG [44] and hyaluronic acid [45], are known to benefit in the management of diabetes, inflammation or diabetic complications. Those metabolites enriched in WLD potentially contribute to the anti-diabetic or anti-inflammatory benefits of WLD in mice receiving HFD.

The anti-diabetic and anti-inflammatory effects of WLD in HFD-fed mice were consistent with previous reports [10]. WLD is relatively more expensive than WHR and it is often used as mixed rice with WHR. The present study added a half-dosage of WLD in a 1:1 mixture with the WHR (WTWD) diet in the dietary regimen to determine the effectiveness of a lower dosage of WLD on metabolism, inflammation, gut microbiota and SCFA production in mice. The results demonstrated that the WTWD diet significantly reduced triglycerides, total cholesterol, insulin resistance, pro-inflammatory cytokines and monocyte adhesion compared to the WHR diet. The beneficial effects on metabolism and inflammation of the WTWD diet were relatively weaker than the WLD diet, as expected. The results suggest that weaker, but significant, metabolic and anti-inflammatory benefits may be achieved using a lower dosage of WLD (representing 10% of daily calorie intake) supplemented with HFD, which may be useful for future regimen design in human trials.

The limitation of the present study includes that the effects of WLD on metabolism, inflammation and gut microbiota were only studied in male, but not in female, mice. Future investigations may be required to compare the effects of WLD in female animals. The present study did not investigate the biological activities of individual components enriched in WLD. The effect and regimen for using WLD in diabetic patients need to be verified in randomized controlled clinical trials.

In conclusion, WLD supplementation in HFD reduced FPG, lipids, insulin, HOMA-IR, monocyte adhesion and pro-inflammation cytokines related to monocyte adhesion compared to that in mice receiving HFD + WHR, which was associated with increased abundances of probiotic bacteria, *L. gasseri,* and propionic acid in feces of mice receiving the WLD diet. The abundance of fecal *L. gasseri* and propionic acid negatively correlated with the metabolic and pro-inflammation cytokines in the mice. The components enriched writing—review and editing fiber. The combination of the findings of the present and previous studies implies that the intake of WLD may improve metabolism and mitigate chronic inflammation in mice through multiple pathways, including, but not limited to, the modulation of gut microbiota, SCFA production and AMPK activation in HFD-fed mice.

## Figures and Tables

**Table 1:** Comparison of macronutrients in WLD and WHR. Sources: U.S. Department of Agriculture [19,20].

	Wild Rice (100 g Raw)	White Rice (Long Strain, 100 g Raw)
Energy (calorie)	357	365
Carbohydrate (g)	74.9	80
Protein (g)	14.7	7.13
Fat (g)	1.08	0.66
Fiber (g)	6.2	1.3

## Data Availability

The data presented in this study are available on request from the corresponding author.

## Abbreviations

AMP	adenosine monophosphate.
AMPA	aminomethylphosphonic acid.
AMPK	AMP-activated protein kinase.
ANOVA	analysis of variance assay.
ELISA	enzyme-linked immunosorbent assay.
FPG	fasting plasma glucose.
G1c-1	6-BP: α-D-Glucose 1, 6-biphosphate.
GlcNAc-1P	*N*-Acetyl-alpha-D-glucosamine 1-phosphate.
Glu-MI	6-(alpha-D-Glucosaminyl)-1D-myo-inositol.
HBSS	Hank’s balanced salt solution.
HFD	high-fat diet.
HOMA-IR	Homeostatic model assessment-insulin resistance.
KAPA	8-amino-7-oxononanoate.
MCP-1	monocyte chemotactic protein-1.
PAI-1	plasminogen activator inhibitor-1.
RGG	α-L-rhamnopyranosyl-(1-2)-β-D-galactopyranosyl-(1)-β-D-glucopyranoside.
SCFA	short chain fatty acid.
TNFα	tumor necrosis factor-α.
WHR	white rice.
WLD	wild rice.
WTWD	13g % of WHR and 13g % of WLD supplemented in HFD.
wt	weight.
